# The House of Commons and the Voluntary Hospitals

**Published:** 1911-07-22

**Authors:** 


### The Hospital

A Journal of

The Medical Sciences and Hospital Administration.

New Series. No. 234, Vol. IX. [No. 1306, Vol. L.] SATURDAY, JULY 22, 1911

### THE HOUSE OF COMMONS AND THE VOLUNTARY HOSPITALS.

We have carefully studied the Insurance Bill now before the House of Commons and its effects on the voluntary hospitals. From the outset we have seen that, as drafted, that Bill must, unless it is amended by the House of Commons, irretrievably injure the welfare of every class of the community throughout the British Islands by dangerously crippling much of the hospital provision on which the sick poor, and, indeed, all sick people, have to depend for their adequate treatment. The more closely the actual figures and facts are considered, as we have examined them, the clearer it becomes that the Insurance Bill, as drafted, unless amended, may destroy the voluntary hospitals in the near future, and at once reduce the present hospital provision for the sick by quite 40 per cent. This statement is made after fully weighing the varying factors of the situation. One factor is the strength of some of the London hospitals, which the King's Hospital Fund Executive has dealt with in a statement published on July 15. We shall show the causes of this supposed strength in figures, and, if well founded in some cases, it will be practically unique so far as the voluntary hospitals are concerned, even in the Metropolis. The case of English, Welsh, Scottish, and Irish voluntary hospitals is quite different, as the figures show. Indeed, the peril to them of this Bill as it stands is grievous and pressing. We hope that the communications on behalf of these hospitals and their supporters which have already reached, or will reach, the English, Welsh, Scottish, and Irish members of the House of Commons will convince them that this is the fact.

This is a financial question, and although we have nothing to do with politics we have much to do with finance, for finance is the bedrock on which the voluntary hospital system depends for its efficiency. Now, a dangerous disregard for business principles characterises the drafting of parts of the Insurance Bill. Clause 8, instead of recognising the voluntary and other hospitals, ignores them altogether, whilst it provides for the creation of sanatoria for patients suffering from tuberculosis and other diseases, as the Local Government Board may approve. Yet this Bill is admittedly based largely upon German legislation, which provides for a guarantee for free treatment and nursing in a recognised hospital, with the means to defray the cost of it. The German plan is however faulty, and must be improved upon. It would appear at first sight, in view of what Mr. Lloyd George has said about the German system, that the omission of our hospitals from the Bill was deliberate. We cannot bring ourselves to believe that this is so, and merely regard it as evidence of a lack of fulness of knowledge of the important hospital financial issues resting upon business bases raised by this Bill, as submitted to the House of Commons. If the House of Commons is to retain the confidence of the people now that it is entirely to control national finance, its members must show that they are men of business of a high order.

We venture to make an appeal to every member of the present House of Commons, and to ask him seriously to consider and weigh an interesting article which we published on the 15th inst. on State Insurance in Germany and how it affects the hospitals. We invite each member's earnest attention to the following facts, which show how the voluntary hospitals in Great Britain and Ireland will be affected by the present Bill, as drafted. We require at least £4,000,000 a year to maintain our voluntary hospitals. These voluntary hospitals are the centre of the research work which is a great national asset, and the effects of that work are of the first importance to every sick person, in whatever state of life he may be found. Mr. Lloyd George has said that he does not wish to interfere with hospital work, but in accepting this sentiment as seriously meant we are obliged to deplore the entire absence of apprehension of the effect of this Bill upon our hospitals which it exhibits. A careful examination of the audited figures of 172 of the principal voluntary hospitals, including the largest and most important hospitals in Great Britain and Ireland, shows to demonstration that Mr. Lloyd George's Bill may reduce the income of the voluntary hospitals in London by not less than 23 per cent., in the provinces by not less than 47½ per cent., in Scotland by at least 45 per cent., and in Ireland by 29 per cent., or a little more. We give these figures for each country because they exhibit the relative financial position of the voluntary hospitals there. Thus, in London about 62 per cent. of the revenue of the voluntary hospitals comes from invested property, the King's Fund contributions, patients' payments and the training and employment of nurses. In the provinces about 32 per cent., in Scotland about 38 per cent., and in Ireland, including grants by local authorities, some 61½ per cent., is derived from the same sources.

These figures show that the voluntary hospitals throughout England and Scotland may be practically paralysed by their loss of revenue, if this Bill passes without providing, as in the case of the German scheme, for an adequate *pro rata* payment for the hospital care and treatment of the insured. The voluntary hospitals of Ireland, which is a poor country, would suffer grievously, and London seriously, though least of all. The latter fact leads us to hope that the London hospital managers will not stand aside, as there seems some inclination in certain quarters to do, but that they will show public spirit and statesmanship by uniting with the English, Scottish, and Irish hospital authorities to bring pressure to bear on the Government, through the House of Commons, that the injury Mr. Lloyd George's scheme will inflict upon the voluntary hospitals may be avoided, and his Bill brought into line with the German plan, by taking power to defray the *pro rata* cost of the hospital treatment the insured may require.

It would be futile for us to appeal to members of the House of Commons without plainly intimating to them how the defects of the Insurance Bill, so far as the voluntary hospitals and hospital treatment are concerned, can be readily redressed. First, Clause 17 should be deleted, because no properly administered voluntary hospital can accept a subscription or donation from an approved society in the circumstances. We are convinced that the amendment of Clauses 8 and 15 will suffice, and that those suggested to Clause 15 alone, if adopted, may provide the necessary safeguards for our voluntary hospitals. In Clause 8 (1) (*b*), page 6, line 7, before the word "sanatoria" to insert the word "hospitals"; and in line 8 to delete the words "tuberculosis or" and the word "other." In line 11 delete the word "sanatorium" and insert "institutional." The amended Sub-Clause (*b*) would then read: "Treatment in hospitals, sanatoria or other institutions when suffering from such diseases as the Local Government Board, with the approval of the Treasury, may appoint. (In this Act called 'institutional benefit.')" In Clause 15 (1), page 15, line 35, for the word "sanatorium" substitute the word "institutional." In line 38 delete the word "a" before the word "sanatoria," and insert the word "hospitals." In line 40, for the word "sanatorium" substitute the word "institutional." Clause 15, as amended, would then read as follows: 15 (1) "For the purposes of administering institutional benefit, local Health Committees shall make arrangements to the satisfaction of the Insurance Commissioners with persons or local authorities having the management of hospitals, sanatoria, or other similar institutions approved by the Local Government Board for the treatment therein of insured persons entitled to institutional benefit under this part of the Act."

We desire members of the House of Commons and all interested in this question to understand that the financial effect of these simple amendments, so far as the hospitals are concerned, need not to any great extent affect the cost of the Bill when it becomes law. What has been possible and practicable in Germany can equally well be introduced into England at the expense of the State. The reason that the proposed amendments may be accepted with wisdom by the Government and the House of Commons is that they will necessarily lead to a thorough reform of the abuses attaching to our existing hospital system, speedily remove such abuses, and so enable every penny of the money available for hospital benefit to be economically expended to the best advantage of everybody concerned. Our suggestions involve co-operation amongst all agencies for hospital care and sick benefit, and the plan upon which this great change, full of promise for the future, making always for efficiency, may be readily carried out, we propose to show in a second article next week. We consider we are entitled to appeal with all earnestness and directness to every member of the House of Commons, because it includes many men who have for years shown the deepest interest in the welfare of the hospitals. We are confident it will be their wish, not only as philanthropists but as statesmen, to provide that the important changes which the Insurance Bill embodies shall be so introduced as to tend to strengthen, and not to destroy, every existing agency for the relief of suffering and the benefit of those least able to provide for themselves in the day of accident or illness.

